# Research Briefs as Communication and Motivation Tools: Knowledge Translation in Medical Education

**DOI:** 10.15694/mep.2017.000183

**Published:** 2017-10-12

**Authors:** Oksana Babenko, Lindsey Nadon, Mao Ding, Lia Daniels

**Affiliations:** 1 University of Alberta

**Keywords:** knowledge translation, communication, motivation, coping

## Abstract

This article was migrated. The article was marked as recommended.

Medical learners are critical stakeholders in medical education research - they are both research participants and end-users of research findings. Traditional forms of disseminating research findings may take years to produce and may never be accessed by participants. Despite this, medical education researchers are responsible for ensuring that research findings reach medical learners faster and more directly. As such, Research Briefs can be a useful vehicle for communicating research findings, rewarding participation in research, and supporting medical learners in their journey to become doctors.

We provide examples of Research Briefs that we have developed to translate knowledge and engage medical learners in our longitudinal research study. We have used Research Briefs to communicate our findings both to participating students and to the larger student community at our university. Doing so has allowed us to start raising awareness of the roles motivation and coping - specifically, achievement goals, self-compassion, and physical activity - play in the learning and well-being of our students.

## Research Briefs

Globally, significant resources and time are invested in the creation of knowledge in health sciences research, including healthcare professional training. ^1- 5^ Despite this, one of the most consistent findings is the failure to translate research into practice and give back to research participants directly. ^1- 5^

In professional education, including medical education, traditional channels and forms of research dissemination (i.e., scientific meetings and peer-reviewed publications) may not necessarily be directly accessible to research participants. We, medical education researchers, are in the position to rectify the situation and ensure that research findings reach our learners in a timely and engaging fashion. In this article, we provide examples of Research Briefs that we have developed for the purpose of knowledge translation and giving back to our participants.

The Research Brief is an evidence-based resource and knowledge translation tool used to communicate research findings directly to groups of people who may not have time, technical knowledge, or access to traditional academic forms of research dissemination (e.g., journal articles, full reports). As such, the Research Brief is a summary of research findings from one or multiple studies on a currently important topic, with a clear take-home message. The Research Brief is not a mini research report or a poster, as it does not include a methodology or sophisticated statistical analyses. It is typically one page long and focuses on providing participants with research results that they may find interesting or relevant. Although there is no “correct” way to design a Research Brief, we recommend minimizing text, usually in the form of bullets or short sentences, and creating graphical elements (e.g., figures, tables, images). Because of this structure, Research Briefs can be created quite quickly and circulated to participants in a timely fashion. Some academic journals employ similar communication tools (e.g., the Last Page) to illustrate concepts, trends, policies, and programs that are important to the academic community, to make the journal’s content more accessible to a wider audience. ^6, 7^

Examples of Research Briefs are shown below. We have used the Research Briefs to communicate research findings to medical students in our university from our longitudinal study, in which students themselves have participated. The study focused on medical students’ motivation and coping strategies. Considering the importance of motivation and coping in the learning process and for the quality of educational outcomes, we aimed to raise students’ awareness of these factors to support our students in their journey to become doctors.

The first Research Brief focuses on the relationships among students’ achievement goals, ^8, 9^ lifelong learning, ^10, 11^ and burnout. ^12, 13^ The second Research Brief focuses on changes in student burnout over the school year, and the effects of engaging in leisure-time exercise on students’ well-being. ^15- 16^ The third Research Brief focuses on the role of self-compassion ^17, 18^ in student burnout and resilience and its relationships with achievement goals.

We have used the Research Briefs to communicate our findings to both participating students and the larger student community at our university via list-serves and the Medical Students’ Association (MSA) online newsletters. ^19, 20^ When we contacted the MSA, the governing body that oversees the interests of all medical students at our university, to help with disseminating the Research Briefs among medical students, our request was well received and the Research Briefs were subsequently included in The Steth, the MSA online newsletter. We take it as an indicator of students’ interest in learning about the roles of motivation and coping with challenges of medical training.

The potential benefits of sharing research results with participants are numerous, including: demonstrating the on-going central nature of the participant’s role in research; providing information that may enhance quality of life and well-being; and raising awareness of the importance and the impact of research on knowledge and practice. ^5^ In the future, Research Briefs may be examined themselves to explore how many participants accessed them, if they are helpful in preventing attrition from longitudinal projects, or if the take-home messages were internalized in a meaningful way. It is our hope that those who conduct research in medical education, and in professional education in general, will lead the way in ensuring that participants receive tangible evidence of the value of their participation in research.

## Take Home Messages

• Take every opportunity to make your research findings accessible to medical learners.
• Research Briefs can help engage medical learners in research and raise their awareness of important learning processes and outcomes.
