# Supplementary material for: Research Briefs as Communication and Motivation Tools: Knowledge Translation in Medical Education
Source: MedEdPublish (2016). 2017 Oct 12;6:183. [Version 1] doi: 10.15694/mep.2017.000183 (PMC10885305; doi:10.15694/mep.2017.000183)
Supplement: Peer review history

# Reviewer report: Reviewer response for version 1

**DOI:** 10.21956/mep.19356.r28303

**Published:** 2019-04-09

**Authors:** Ken Masters

**Affiliations:** 1 Sultan Qaboos University

This review has been migrated. The reviewer awarded 4 stars out of 5 An interesting, short (brief :-) paper on the concept of Research Briefs to disseminate research information in medical education.Yes, I can certainly see that these would have an important role in disseminating the essence of a piece of research. Perhaps one added suggestion to the Research Brief, however: if it is reporting of a piece of research (or several), then it supplies links or references to the research on which is based (if possible). There are few things more infuriating than brief reports that refer to other research but do not indicate the source of that research.

Reviewer Expertise:

NA

No decision status is available

# Reviewer report: Reviewer response for version 1

**DOI:** 10.21956/mep.19356.r28305

**Published:** 2017-11-01

**Authors:** Iriagbonse Osaigbovo

**Affiliations:** 1 N/A

This review has been migrated. The reviewer awarded 4 stars out of 5 This is a commendable initiative that Should be adopted by all Med Ed scholars. It has given me the idea to give a feedback to the students whom I recently conducted a survey with.

Reviewer Expertise:

NA

No decision status is available

# Reviewer report: Reviewer response for version 1

**DOI:** 10.21956/mep.19356.r28304

**Published:** 2017-10-17

**Authors:** Gerard Flaherty

**Affiliations:** 1 National University of Ireland Galway

This review has been migrated. The reviewer awarded 4 stars out of 5 I welcome this contribution to the health professions education literature. It clearly describes a novel initiative to increase the involvement of students in the research process by feeding back key findings to them of research projects in which they participated. This may serve to increase student interest in educational research and improve response rates in future studies. The rationale for the study is sound and the info-graphical presentation of the Research Briefs is appropriate for their intended purpose. I wonder if students would welcome some deeper insight into the statistical certainty of the results, however, even to serve as an exemplar of good research practice.

Reviewer Expertise:

NA

No decision status is available

# Reviewer report: Reviewer response for version 1

**DOI:** 10.21956/mep.19356.r28306

**Published:** 2017-10-12

**Authors:** Nandalal Gunaratne

**Affiliations:** 1 Faculty of Medicine, Wayamba University of Sri Lanka

This review has been migrated. The reviewer awarded 4 stars out of 5 In an era where visual arts and visual literacy is becoming manifest in education and social media, this approach is likely to become more and more acceptable.With online resources for creation of infographics, creating research Briefs and updated information on care is easy.This paper confirms a user case to bring student views back to them.A step in the right direction.

Reviewer Expertise:

NA

No decision status is available
